# Candidate genes for first flower node identified in pepper using combined SLAF-seq and BSA

**DOI:** 10.1371/journal.pone.0194071

**Published:** 2018-03-20

**Authors:** Xiaofen Zhang, Guoyun Wang, Bin Chen, Heshan Du, Fenglan Zhang, Haiying Zhang, Qian Wang, Sansheng Geng

**Affiliations:** 1 Beijing Vegetable Research Center, Beijing Academy of Agriculture and Forestry Sciences, Key Laboratory of Biology and Genetic Improvement of Horticultural Crops (North China), Ministry of Agriculture, Beijing, P.R. China; 2 College of Horticulture, China Agricultural University, Beijing, P.R. China; Louisiana State University College of Agriculture, UNITED STATES

## Abstract

First flower node (FFN) is an important trait for evaluating fruit earliness in pepper (*Capsicum annuum* L.), but the genetic mechanisms that control FFN are still poorly understood. In the present study, we developed 249 F_2_ plants derived from an intraspecific cross between the inbred pepper lines Z4 and Z5. Thirty plants with the highest FFN and 30 plants with the lowest FFN were chosen and their DNAs were pooled according to phenotype to construct two bulked DNA pools. Specific-locus amplified fragment sequencing (SLAF-seq) was combined with bulked segregant analysis (BSA) to identify candidate regions related to FFN. According to our genetic analysis, the FFN trait is quantitatively inherited. A total of 106,848 high-quality single nucleotide polymorphism (SNP) markers were obtained, and 393 high-quality SNP markers associated with FFN were detected. Ten candidate regions within an interval of 3.98 Mb on chromosome 12 harboring 23 candidate genes were identified as closely correlated with FFN. Five genes (*CA12g15130*, *CA12g15160*, *CA12g15370*, *CA12g15360*, and *CA12g15390*) are predicted based on their annotations to be associated with expression of the FFN trait. The present study demonstrates an efficient genetic mapping strategy and lays a good foundation for molecular marker-assisted breeding using SNP markers linked to FFN and for cloning and functional analysis of the key genes controlling FFN.

## Introduction

Pepper (*Capsicum annuum* L.), Solanaceae, has a sympodial shoot structure with a solitary flower known as the first flower. During vegetative growth, the pepper shoot apical meristem (SAM) produces stems and leaves that are arranged in an alternate spiral pattern. The SAM can later undergo a transition to an inflorescence meristem that subsequently develops into the first flower at start of the transition from vegetative to reproductive growth [1, 2]. Therefore, the formation of the first flower is a crucial phase in plant growth that is regulated by a complex network of genes that promote [1–6] or suppress [7, 8] flowering. The *FASCICULATE* (*FA*) and *CaJOINTLESS* (*CaJ*) genes control sympodial shoot development in pepper. *FA* also stimulates late flowering [4] and *CaJ* promotes first flower development while suppressing inflorescence development in pepper [1]. The floral meristem identity genes *Ca-ANANTH*, *Ca-LEAFY*, and *Capsicum annuum* S promote flower formation [2, 3], while *CaBLIND* and *CaHAM* regulate axillary branching [5, 6]. Additionally, *CaJ* and *CaBLIND* are both epistatic to *FA* for controlling flowering time and suppressing vegetative growth during the reproductive phase of pepper [1, 5]. However, the *CaRNA*-binding protein has a repressive effect on the flowering time [8], and *Ca-APETALA2*, which maps to pepper chromosome 02, represses flowering in pepper [7]. Although these genes reportedly control the transition from the vegetative stage to the flowering stage during development in pepper, the molecular regulatory mechanisms controlling the formation of the first flower in pepper are still poorly understood.

The node at which the first flower develops has been designated as the first flower node (FFN) in pepper. The position of this node, or its node number, on the primary axis from the cotyledonary node to the first flower node defines the FFN trait. Pepper species exhibit extensive natural variation in FFN [9, 10]. Thus, FFN has been an important trait for evaluating fruit earliness in pepper breeding [11]. In general, earliness in pepper has also been described in reference to some other agronomic traits such as flowering date or flowering earliness [12, 13], plant height [10], FFN [9, 10, 11], the number of leaves on the primary axis [12, 14], the number of lateral branches on the primary axis [15]. For example, Liu (2015) found that plants with lower FFN and moderate plant height exhibited earlier maturity [10]. Furthermore, FFN is correlated positively with plant height, main stem length, the number of leaves, and the number of branches [10], which are controlled by quantitative trait loci (QTL). In addition, FFN is also a primary factor controlling flowering time [16]. In pepper, QTL controlling flowering date or flowering earliness have been detected on pepper chromosomes 02, 04, and 12 [12, 13]; QTLs for primary axis length have been identified on pepper chromosomes 02, 03, 09, and 12 [12, 13]; QTLs for plant height have been detected on pepper chromosomes 02–08 [15, 17, 18]; QTLs for the lateral branch number on the primary axis have been identified on chromosome 02 [15]; and QTLs for the number of leaves on the main stem have been detected on all pepper chromosomes except for chromosome 09 [12, 13, 14, 19]. In tomato, FFN has been mapped to tomato chromosomes 02, 03, 05b, 08b, and 11 [20]. However, studies to map QTLs and identify genes that control the FFN trait in pepper have been limited to date. The publication of the pepper genome sequence in 2014 [21] should facilitate finely mapping of the FFN trait in pepper.

Bulked segregant analysis (BSA) [22] is a simplified strategy for identifying molecular markers tightly linked to a gene. In BSA, a pair of bulked DNA samples is derived by pooling DNAs from individuals that are grouped according to contrasting extreme phenotypes; these bulked DNAs are then genotyped. In pepper, loci controlling specific traits have been identified using a combination of BSA and various molecular markers [16, 23–25]. However, it is challenging to develop thousands of candidate molecular markers and screen them in the bulked pools to discover the small subset of markers diagnostic for the target phenotype. Next-generation sequencing has promoted the development of new strategies to leverage the advantages of BSA. For example, specific-locus amplified fragment sequencing (SLAF-seq) is a strategy for discovery of single nucleotide polymorphisms (SNPs) facilitated by reduced-representation genome sequencing and next-generation sequencing technologies. SLAF-seq is a rapid, high-throughput, high-accuracy, and cost-effective strategy for large-scale SNP discovery and genotyping [26]. The combined BSA and SLAF-seq strategy has been used for SNP discovery in many species including rice [27], cotton [28], *Brassica napus* [29], and melon [30], among others. Integrated BSA and SLAF-seq strategies also have been successfully used for SNP discovery in pepper to refine the region containing a gene for resistance to *Phytophthora* root rot to a 2.57-Mb region [31]. This combined approach has also been used to identify one major QTL associated with resistance to *Cucumber mosaic virus* on pepper chromosome 02 [32]. These studies have demonstrated the efficiency of combined BSA and SLAF-seq as a strategy for the identification of genes or QTLs linked to a specific trait in plants.

Thus, in the present study we have used a combined BSA and SLAF-seq strategy to identify genomic regions linked to the FFN trait in DNAs pooled from distinct FFN phenotypes in an F_2_ population derived from a cross between inbred parental lines Z4 and Z5 in pepper. Our objectives were to: 1) investigate the mode of inheritance of the FFN trait in pepper; 2) identify the genomic regions correlated with variation in FFN; and 3) identify candidate genes and SNP markers linked to the FFN trait in pepper.

## Materials and methods

### Plant materials

The first flower node was measured as the number of nodes on the primary axis from the node of the cotyledon to that of the first flower. An F_2_ mapping population comprising 249 plants was derived from a cross between the *Capsicum annuum* pepper lines Z4 and Z5, which were bred in Beijing Vegetable Research Center of the Beijing Academy of Agriculture and Forestry Sciences. Plants from line Z5 tend to be tall and have a high FFN (i.e., 19 nodes), while those from line Z4 tend to be of medium height with a low FFN (i.e., 9 nodes). Parental and F_2_ plants were grown in the greenhouse at the Beijing Vegetable Research Center of the Beijing Academy of Agriculture and Forestry Sciences in Beijing, China.

### Genetic analysis of the FFN trait

Phenotypic data were statistically analyzed using Microsoft Excel (Microsoft Office, Microsoft, 2003) and data were plotted using SigmaPlot 10.0 (SPSS Inc., Chicago, IL).

### DNA extraction and construction of DNA pools

Total genomic DNA was isolated from young leaves of the both parental lines and F_2_ plants using the cetyl trimethyl ammonium bromide (CTAB) method [33]. Two DNA pools were constructed by separately pooling an equal amount of DNA from each of 30 extreme high FFN plants (H-pool) or 30 extreme low FFN plants (L-pool) identified in the F_2_ population.

### Construction of SLAF libraries and high-throughput sequencing

The genome of *Capsicum annuum* cv. ‘Criollo de Morelos 334’ (CM334) (http://peppergenome.snu.ac.kr/download.php, version 1.55) was used as the reference genome in the present study. Genomic DNAs from both parents and the H-pool and the L-pool were digested with the restriction enzyme *Hae*III (New England BioLabs, Ipswich, USA) after optimizing restriction enzyme digestion completeness to obtain even genome coverage. Single-nucleotide A overhangs were polished from these DNA fragments using Klenow fragment (New England BioLabs), and fragments were then ligated to dual-index sequencing adaptors [34]. Adaptor-ligated fragments were then amplified by PCR, purified, pooled, and screened to construct the SLAF library. Details of the processes for SLAF library construction and screening were performed as described in Sun et al. (2013) [26]. Target DNA fragments of sizes in the range of 414–514 bp were selected as SLAFs from the quality-tested library and prepared for paired-end sequencing on an Illumina HighSeq 2500 platform (Illumina, Inc., San Diego, CA, USA) at Beijing Biomarker Technologies Corporation in Beijing, China (http://www.biomarker.com.cn). To check the reliability and validity of sequencing and screening processes, the genome of rice (*Oryza sativa* L. *japonica*, http://rice.plantbiology.msu.edu/, version 7.0) was selected as a control to undergo in parallel the same library construction and sequencing processes as performed for the pepper mapping population.

### Data analysis for SLAF-seq

Raw reads were filtered for quality and trimmed to remove adaptors, and then sequence quality was assessed based on sequencing quality scores and guanine-cytosine (GC) content [26]. The proportion of sequencing quality scores ≥Q30 in the four libraries was >80% (A quality score of Q30 indicates a 0.1% error rate or 99.9% sequence accuracy.). High-quality reads were mapped onto the pepper reference genome using BWA software [35]. We clustered all paired-end reads that had perfect index reads according to sequence similarity among both parents and the two pooled libraries using blast [36]. Sequences with >90% identity were grouped into a single SLAF locus (or SLAF tag).

### Identification of high-quality SNPs

Single-nucleotide polymorphisms (SNPs) were detected primarily using GATK software [37]. Using clean reads mapped onto the reference genome, local realignments were conducted, and SNPs were detected using GATK software as described by https://www.broadinstitute.org/gatk/guide/best-practices.php. To ensure the accuracy of SNPs identified using GATK, SAMtools software also was used to detect SNPs [38]. The intersection of SNPs that were detected using both GATK and SAMtools software was designated as final SNPs for further analysis. In addition, the localization (e.g., upstream, downstream, or intergenic regions) of SNPs, and the coding effects (e.g., synonymous or non-synonymous mutation) of SNPs were annotated using SnpEff software [39] based on gene model annotations at the pepper reference genome databases (http://peppergenome.snu.ac.kr/download.php, version 1.55). Before performing association analysis, SNPs were filtered using the following criteria: SNPs with multiple alleles were filtered out; SNPs with sequencing depths of less than 4× in each pool or parent were excluded; SNPs with the same genotypes among pools were removed; and SNPs with recessive alleles that were not inherited from parents with recessive genotypes in pools were filtered out. Ultimately, a collection of high-quality SNP markers was obtained for use in association analysis.

### Association analysis

Association mapping was performed using either Euclidean distance (ED) [37] or the SNP-index algorithm [40, 41] separately. ED between the allele frequencies at each SNP in the L-pool and H-pool was calculated as in Hill et al. (2013) using the equation [37]

$$ED=\sqrt{{\left(A_{L}{}_{-pool}-A_{H-pool}\right)}^{2}+{\left(C_{L-pool}-C_{H-pool}\right)}^{2}+{\left(G_{L-pool}-G_{H-pool}\right)}^{2}+{\left(T_{L-pool}-T_{H-pool}\right)}^{2}},$$

where each letter (*A*, *C*, *G*, *T*) indicates the frequency of its corresponding DNA nucleotide. ED values were then squared to decrease the effects of noise and increase the effects of large ED measurements based on distance measurements raised to a power (ED^x^). Data were then fitted using Loess regression [37]. In addition, the threshold for the significance of marker-trait associations was set at 1% of the biggest Loess-fitted values. The genomic regions at which the Loess-fitted values exceeded the threshold were then designated as candidate regions related to FFN in pepper.

The SNP-index algorithm is valuable for finding significant differences in the frequencies of genotypes between DNA pools. The frequency of genotypes in the SNP-index algorithm is denoted by SLAF depth [42]. The SNP-index is calculated as the proportion of the depth of L-pool or H-pool derived from the female parent relative to the two parental depths, and then the ΔSNP-index is defined by subtracting the H-pool SNP-index from the L-pool SNP-index. Therefore, the ΔSNP-index is equal to 0 if the SNP-index of the L-pool is equal to that of the H-pool. If the ΔSNP-index value equals 1, one genotype is associated almost entirely with the high FFN pool, and the associated SNPs are therefore linked closely to the high FFN phenotype. On the contrary, if the ΔSNP-index value equals -1, the associated SNPs are linked to the low FFN phenotype. The confidence coefficient of the ΔSNP-index was calculated, and all ΔSNP-index values were fitted using SNPNUM [38]. FFN-related regions were identified when the fitted values of markers were above the threshold at the 99% of confidence interval.

Finally, ED, SNP-index, and ΔSNP-index values were plotted and the intersections between the candidate regions for FFN that were identified using the ED and SNP-index methods were designated as the final candidate FFN-related regions. A circular graph representing the distribution of chromosomes, genes, SNPs, ED values, and ΔSNP-index values was then plotted using CIRCOS 0.66 software (http://circos.ca/).

### Annotation of candidate genes

To verify the predicted genes in the target region, we compared these candidate genes to the CM334 reference genome using blast. Putative functions of candidate genes were predicted based on sequence alignments with annotated genes in the databases at Swiss-Prot (http://www.uniprot.org/), the Gene Ontology (GO, http://www.geneontology.org/), the Cluster of Orthologous Groups of proteins (COG, http://www.ncbi.nlm.nih.gov/COG/), the Kyoto Encyclopedia of Genes and Genomes (KEGG, http://www.genome.jp/kegg/), and the NCBI non-redundant protein database (NR, ftp://ftp.ncbi.nih.gov/blast/db/) using blastp with default parameters.

## Results

### Genetic analysis of the FFN trait

**Fig 1:**
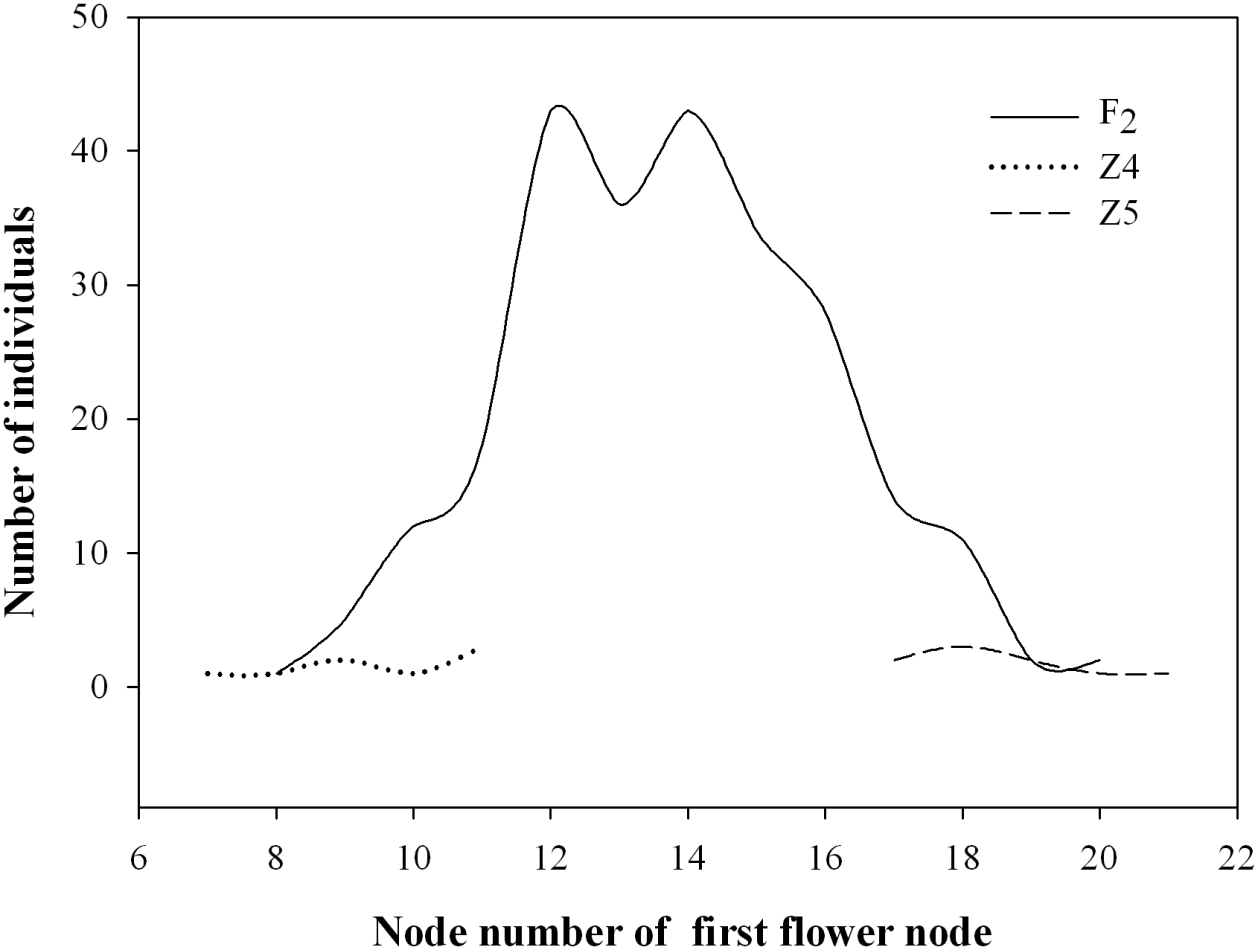
The phenotypic distribution of the first flower node (FFN) trait in the F_2_ population and inbred pepper lines Z4 and Z5.

An F_2_ population comprising 249 plants developed from the Z4 × Z5 cross was used to investigate the inheritance of FFN, which ranged in value from 8 to 20 (Fig 1). The continuous phenotypic distribution and transgressive segregation of FFN in the F_2_ population in Fig 1 suggests that FFN is quantitatively inherited, and predicts that the FFN phenotype is likely controlled bysome major genes and some minor genes.

### Evaluation of the SLAF library

*Hae*III was chosen as the restriction enzyme for SLAF library construction based on preliminary experiments to identify a restriction enzyme that produced SLAFs evenly distributed on the pepper reference genome (S1A Fig). The SLAF libraries were then evaluated to verify the accuracy of sequencing compared with the rice genome control. First, the sequence reads of the rice genome control were compared with those of the pepper reference genome using BWA software [35]. The percentage of mapped paired-end reads in the pepper genome was 80.12% (Table 1), which is a typical efficiency for mapped paired-end reads from a SLAF library. Second, the efficiency of enzyme digestion is an important index for revaluating the likelihood of successful SLAF experiments. More efficient restriction enzyme digestion results in more successful SLAF experiments. In the present study, the efficiency of restriction enzyme digestion was 91.45% (Table 1), indicating that restriction enzyme digestion was adequate for constructing the SLAF libraries. Finally, average read length of SLAF was determined based on the insert size distribution of mapped paired-end reads in the rice genome. When the distance between reads at the ends of SLAFs was within 1 Kb, the integrity of SLAFs was greater than 0.1 and sequencing depth was greater than 3×, SLAFs of 414–514 bp in length were used for further analyses (S2 Fig).

**Table 1:** The efficiency of mapped paired-end reads and the efficiency of *Hae*III restriction enzyme digestion in the control genome.

**The efficiency of mapped reads**	**(%)**
Mapped paired-end reads	80.12
Mapped single-end reads	6.10
Unmapped reads	13.78
**Efficiency of enzyme digestion**	**(%)**
Complete digestion	91.45
Partial digestion	8.55

### Sequence data analysis and SNP identification

After constructing SLAF libraries and performing high-throughput sequencing, a total of 153,821,146 raw reads were obtained with an average read length of 100 bp (Table 2). After filtering reads, 76.24 Mb of clean reads and 15.25 Gb of clean bases remained. The average proportion of reads with Q30 scores was 92.11% and average GC content was 39.17%, indicating that most bases were of high quality. Further, BWA software was used to map clean reads to the pepper reference genome [35]. The proportion of clean reads that could be mapped to the reference genome was >88.81%, which also reflects sequencing accuracy (Table 2). In total, cluster analysis identified 492,259 SLAFs (S1 Table) that were distributed evenly on the chromosomes of the pepper reference genome (S1B Fig). The highest number of SLAFs occurred on pepper chromosome 03, while the lowest number of SLAFs occurred on chromosome 08. There were 429,868 SLAFs in Z4, 420,488 in Z5, 476,558 in the L-pool, and 477,116 in the H-pool. Average sequencing depths were 36.92× in the parental libraries, 32.36× in the L-pool, and 36.57× in the H-pool (Table 2).

**Table 2:** Summary of sequence data from parental line DNAs and bulked DNA pools.

DNA sample	Raw reads	Clean reads	Clean bases	Q30 (%)	GC (%)	Proportion of mapped reads (%)	Number of SLAFs	Average depth	SNP coverage
Z4	36,393,690	18,196,845	3,639,369,000	92.08	39.49	91.30	429,868	36.66	0.042
Z5	36,455,620	18,227,810	3,645,562,000	92.20	38.32	90.44	420,488	37.18	0.043
L-pool	37,789,236	18,894,618	3,778,923,600	92.08	39.64	88.81	476,558	32.36	0.047
H-pool	41,848,984	20,924,492	4,184,898,400	92.06	39.24	90.07	477,116	36.57	0.047

L-pool, the DNA pool from plants with the lowest first flower node phenotype; H-pool, the DNA pool from plants with the highest first flower node phenotype; Proportion of mapped reads (%), clean reads mapped to the pepper reference genome as a percentage of the total clean reads; Q30, a quality score of 30 indicates 0.1% error rate or 99.9% sequence accuracy; GC, guanine-cytosine content; SLAF, specific-locus amplified fragment; SNP, single-nucleotide polymorphism.

A total of 1,001,405 SNPs were identified using GATK [37] and SAMtools software [38], and the properties of total SNPs were showed in S2 Table. The number of SNPs on each chromosome (shown in S1 Table) ranged from 21,371 on chromosome 08 to 150,965 on chromosome 11. The sequence coverage of the pepper genome was more than 0.042 when calculated across all markers. The distribution of SNPs on each pepper chromosome is shown in S1C Fig. After filtering, a total of 106,848 of high-quality SNPs remained as useful markers for association analysis to identify candidate regions associated with FFN in pepper.

### Association analysis based on Euclidean distance

**Fig 2:**
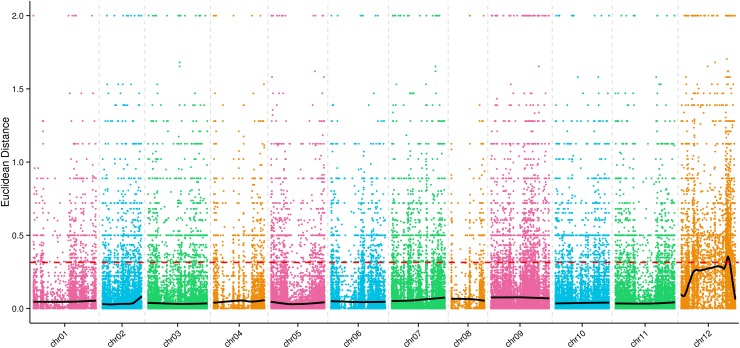
Graph of Euclidean distance-based association values between SNPs on each chromosome. The *x*-axis represents the 12 pepper chromosomes, and the *y*-axis represents the association value based on Euclidean distance. The colored dots represent the association values based on Euclidean distance at each SNP location. The red dashed line and black line represent association threshold and Loess-fitted values, respectively. Higher association values based on Euclidean distance indicate stronger association between a SNP and first flower node (FFN).

ED values were calculated for total of 106,848 high-quality SNP markers to identify genomic regions and markers associated with FFN. The ED value for each pair of SNPs was calculated. ED values were squared to decrease the effects of noise, and association values were then fitted by Loess regression [37]. The marker-trait association threshold was set to 0.31based on the 1% of the biggest Loess-fitted values. A graph was generated for the association values based on ED for each chromosome (Fig 2). Only one candidate region related to FFN for which the Loess-fitted value was above the threshold was detected on chromosome 12. In this region, we identified an interval from 196,328,926 to 210,751,601 bp containing 125 genes, among which two genes contained non-synonymous SNPs (Table 3). There were also 1,069 high-quality SNP markers within these candidate regions.

**Table 3:** Association information obtained *via* SNP-index or Euclidean distance.

ChrID	Start (bp)	End (bp)	Size (Mb)	Number of SNP marker	Number of gene
**Euclidean distance**					
Chr12	196,328,926	210,751,601	14.42	1,069	125
**SNP-index**					
Chr12	198,792,663	198,793,573	0.00091	3	0
Chr12	199,120,000	199,293,531	0.17	4	1
Chr12	199,293,569	199,952,426	0.66	58	12
Chr12	199,970,974	200,210,994	0.24	21	1
Chr12	200,211,315	200,281,965	0.071	4	1
Chr12	200,326,950	200,359,194	0.032	3	0
Chr12	200,629,900	200,638,585	0.0087	3	0
Chr12	201,010,194	201,036,653	0.026	6	0
Chr12	201,044,155	201,044,589	0.00043	5	0
Chr12	201,055,482	203,828,248	2.77	286	8

ChrID, the abbreviation of chromosome followed by a chromosome number; SNP, single-nucleotide polymorphism.

### Association analysis based on SNP-indices

**Fig 3:**
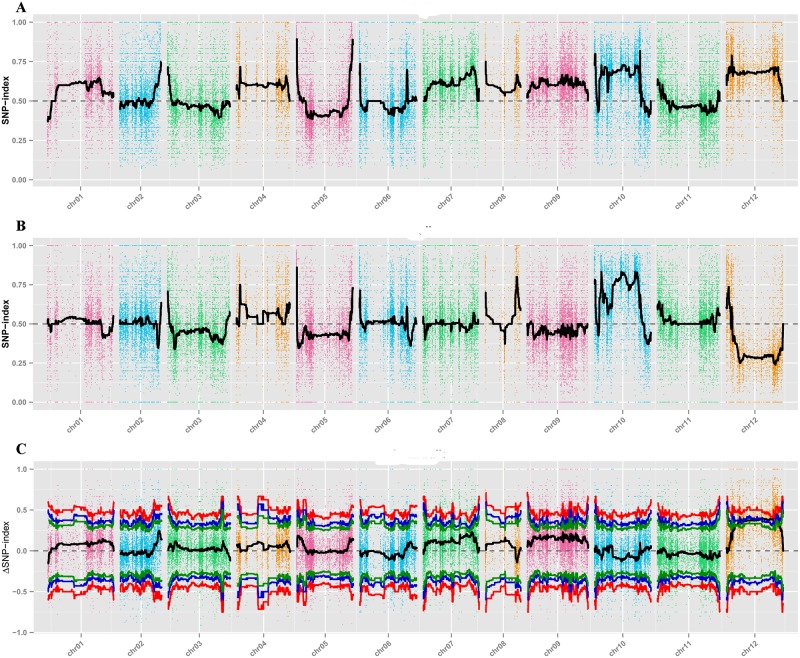
Graphs of the SNP-index of the H-pool (A), the L-pool (B), and the ΔSNP-index values (C) for association analysis. The *x*-axis and *y*-axis indicate the 12 pepper chromosomes and the SNP index, separately. The black line represents the fitted SNP-index or ΔSNP-index. The red, blue, or green line indicates the threshold for association with FFN at the 99%, 95%, or 90% confidence interval, respectively.

The SNP-indices for 106,848 SNP markers were calculated, and graphs of SNP-index values were drawn for the H-pool (Fig 3A) and L-pool (Fig 3B) as plots of average SNP-index values against each sliding-window position in the CM334 genome assembly. The ΔSNP-index was determined by subtracting the SNP-index of the L-pool from that of the H-pool, and was plotted (Fig 3C). Ten candidate regions for control of the FFN phenotype were identified on chromosome 12 by examining the ΔSNP-index plot and identifying the fitted values of SNP markers that were above the threshold at the 99% confidence interval. Although we did not identify any candidate genes within the five FFN candidate regions, in five other regions we identified one gene within the interval from 199,120,000 to 199,293,531 bp, one gene within the interval from 199,970,974 to 200,210,994 bp, one gene within the interval from 200,211,315 to 200,281,965 bp, 12 genes within the interval from 199,293,569 to 199,952,426 bp, and eight genes within the interval from 201,055,482 to 203,828,248 bp. A total of 393 SNP markers were detected within those candidate regions within a 3.98 Mb interval (Table 3). However, none of the identified genes carried non-synonymous SNPs.

Final candidate regions tightly associated with the FFN trait in pepper were identified using a combination of ED and SNP-index association analysis. The same candidate regions were identified by ED and SNP-index association analyses within the interval from 0.00091 to 2.77 Mb on chromosome 12 that harbors 23 genes.

### Visualization of the combined results of SLAF-seq and BSA

We used a strategy combining SLAF-seq and BSA to identify genomic regions, SNPs, and genes associated with FFN. All of the results of these approaches are visualized in the circular graph shown in S3 Fig. The circles in this graph, from the outermost to the innermost, represent the 12 pepper chromosomes, the distribution of genes on the pepper chromosomes, SNP density, ED values, and ΔSNP-index values, respectively.

### Annotated SNP markers and genes within the candidate region

We identified a total of 393 high-quality SNP markers within the candidate regions (Table 3). There were 58 high-quality SNP markers within the interval from 199,293,569 to 199,952,426 bp, and three high-quality SNP markers within each of the intervals from 198,792,663 to 198,793,573 bp, from 200,326,950 to 200,359,194 bp, and from 200,629,900 to 200,638,585 bp, respectively (Table 3). The localizations and coding effects of SNPs in these candidate regions were annotated using SnpEff software [39]. There were 3, 588, and 3 SNPs between genes in the Z4 and Z5 genomes in their upstream, intergenic, and downstream regions, respectively. We also identified 4, 418, and 1 SNPs between the H-pool and the L-pool in the upstream, intergenic, and downstream regions of genes, respectively (S3 Table). However, no non-synonymous SNPs were identified in the candidate regions among parental lines and both pools (S3 Table). Thus, more than 98% of SNP markers were located in intergenic regions.

**Fig 4:**
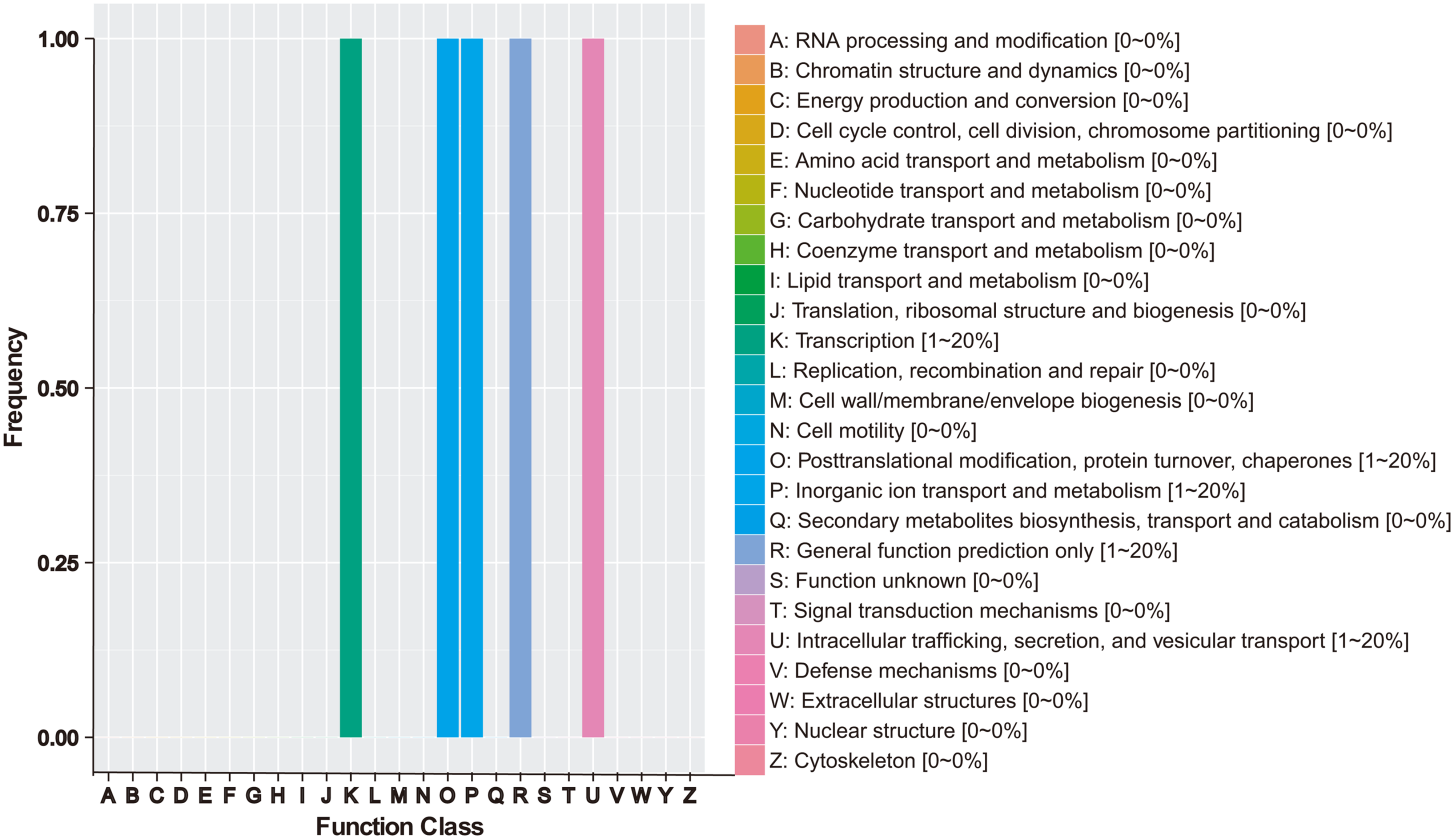
Functional classification of candidate genes at the Cluster of Orthologous Groups of proteins database.

We predicted that 23 protein-coding genes (Table 4), including nine genes with no annotations in the public databases, are be located within the 10 candidate regions for FFN on pepper chromosome 12, based on the current annotation of the CM334 pepper reference genome. We found annotations for 12, 5, 1, and 12 genes in the candidate regions in the GO, COG, KEGG, and Swiss-Prot databases, respectively. Some genes were annotated with more than one term in different domains, and could thus be categorized into two or more functional categories (S4 Fig). GO term enrichment analysis of predicted genes yielded functional assignments of 45 genes to the ‘cellular component’ domain, 12 genes to the ‘molecular function’ domain, and 43 genes to the ‘biological process domain (S4 Fig). As shown in S4 Fig, 11 genes in the ‘cellular component’ domain are associated with the GO term ‘cell part’ (GO:0044464); four genes in the ‘molecular function’ domain are associated with the GO term ‘catalytic activity’ (GO:0003824), four genes in the ‘molecular function’ domain are associated with ‘transporter activity’ (GO:0005215), four genes in the ‘molecular function’ domain are associated with ‘binding’ (GO:0005488); and 10 genes are associated with the GO term ‘cellular process’ (GO:0009987) in the ‘biological process’ domain. COG analysis predicted five genes, among which the functions of *CA12g15090*, *CA12g15100*, and *CA12g15320* were related to ‘transcription’, ‘general function prediction only’, and ‘inorganic ion transport and metabolism’, respectively. The function of *CA12g15130* was related to ‘intracellular trafficking, secretion, and vesicular transport’, and that of *CA12g15370* was associated with ‘posttranslational modification, protein turnover, and chaperones’ (Table 4; Fig 4). KEGG analysis identified a match for only one gene (*CA12g15130*), a homolog of *SEC22* in *Arabidopsis thaliana* that encodes a 25.3 kDa vesicle transport protein that takes part in both the ‘phagosome’ pathway (S5 Fig) and the ‘SNARE interactions in the vesicular transport’ pathway (S6 Fig). Five of these 23 candidate genes might be related to first flower node development based on our current annotation, but their functions must be further studied. *CA12g15130* plays an important role in vesicle trafficking from the endoplasmic reticulum to the Golgi complex, and is highly expressed in *Arabidopsis* flowers [43]. *CA12g15360*, which encodes the pentatricopeptide repeat-containing protein At2g40720, was predicted to take part in xylem and phloem pattern formation according to its GO annotation. The RING-H2 finger protein ATL52 (*CA12g15370*) might be involved in the protein ‘ubiquitination’ pathway. Hu et al. (2003) reported that ATL52 is expressed in *Arabidopsis* flowers [44]. Additionally, *CA12g15390* matched a purine permease 1, which is highly expressed in leaves, stems, and flowers, but not in roots [45, 46]. This protein has been predicted to function as a transporter for nucleotides, nucleosides, and their derivatives [45]. *CA12g15160* is homologous to *At1g48120* from *Arabidopsis*, which encodes a serine/threonine-protein phosphatase 7 long form homolog that is expressed in the SAM, root tips, hydathodes, leaf vasculature, and mature flowers [47]. Because the stem and leaves arise from the SAM, this gene might be important for the development of the first flower node, as it might be required to maintain cell division activity in meristematic cells. These five predicted genes that are expressed in flowers, xylem and phloem, stem, and the SAM [43–47] participate in many important biological processes in relevant tissues and organs and might play important roles in the FFN trait in pepper. The molecular functions, if any, of these candidate genes in the control of FFN need to be further examined.

**Table 4:** Annotations of 23 candidate genes for first flower node (FFN) identified on chromosome 12 of pepper.

Gene ID	Annotation	Database
*CA12g15070*	Uncharacterized protein LOC101248504	NR
*CA12g15080*	Uncharacterized protein LOC101266104	NR
*CA12g15090*	DNA-directed RNA polymerase subunit beta	COG, GO, Swissprot, NR
*CA12g15100*	DUF21 domain-containing protein At1g47330	COG, GO, Swissprot, NR
*CA12g15110*	Transmembrane protein 87A	GO, Swissprot, NR
*CA12g15120*	Uncharacterized protein LOC101260122	NR
*CA12g15130*	25.3 kDa vesicle transport protein	COG, GO, KEGG, Swissprot, NR
*CA12g15140*	Uncharacterized protein LOC101267004	NR
*CA12g15150*	Serine-rich adhesin for platelets	Swissprot, NR
*CA12g15160*	Serine/threonine-protein phosphatase 7 long form homolog	Swissprot, NR
*CA12g15170*	Uncharacterized protein LOC101264603	NR
*CA12g15180*	Hypothetical protein	GO, NR
*CA12g15190*	Uncharacterized protein LOC101246698	NR
*CA12g15210*	Uncharacterized protein LOC101267316	NR
*CA12g15220*	Uncharacterized protein LOC101244351	NR
*CA12g15320*	Potassium transporter 11	COG, GO, Swissprot, NR
*CA12g15330*	Enzymatic polyprotein-like	GO, NR
*CA12g15340*	Predicted protein	NR
*CA12g15350*	Aquaporin NIP1-3	GO, Swissprot, NR
*CA12g15360*	Pentatricopeptide repeat-containing protein At2g40720	GO, Swissprot, NR
*CA12g15370*	RING-H2 finger protein ATL52	COG, GO, Swissprot, NR
*CA12g15380*	Blue copper protein	GO, Swissprot, NR
*CA12g15390*	Purine permease 1	GO, Swissprot, NR

GO, Gene Ontology; COG, Cluster of Orthologous Groups of proteins; KEGG, Kyoto Encyclopedia of Genes and Genomes; NR, NCBI non-redundant protein database.

## Discussion

### SLAF-seq and BSA has been successfully used in many gene mining research

SLAF-seq, a recently developed strategy for SNP discovery and large-scale genotyping [26], has been applied successfully for construction of linkage maps and analysis of QTL in many species [48–52]. Compared with earlier methods of marker development (e.g., restriction fragment length polymorphism (RFLP), random amplified polymorphic DNA (RAPD), amplified fragment length polymorphism (AFLP), and simple sequence repeat (SSR), SLAF-seq has several advantages including high accuracy, high throughput, efficiency, and cost effectiveness for SNP discovery and large-scale genotyping [26]. SLAF-seq takes advantage of large amounts of sequence data to develop the SLAF markers, improve marker development, and increase coverage of entire genomes [48]. In addition, BSA is an efficient way to identify markers that are specific for a trait of interest. We combined the SLAF-seq and BSA techniques to identify the genomic regions associated with the FFN trait in pepper. Previous studies have combined the SLAF-seq and BSA approaches successfully in many organisms [27–32]. For example, Xu et al. (2016) combined SLAF-seq and BSA to map resistance to *Phytophthora* root rot to pepper chromosome 10 [31]. Guo et al. (2017) have also identified one major QTL linked to *Cucumber mosaic virus* resistance in the physical interval from 152.87 to 153.20 Mb on chromosome 02 in pepper using this integrated strategy [32].

### We detected 10 FFN candidate regions efficiently by using SAF-seq combined with BSA

We obtained a total of 76.24 Mb of clean reads from 153.82 Mb of raw reads with an average read length of 100 bp, and evaluated sequencing depth and sequencing quality scores. For successful SLAF-seq, Sun et al. (2013) suggested that sequencing depth should be greater than 6× and quality scores should not be lower than Q30 [26]. The restriction enzyme digestion efficiency, sequencing depths, Q30 scores, and percentages of mapped paired-end reads achieved in our study demonstrate that the construction and sequencing of our SLAF library were sufficiently efficient, accurate, and high-quality.

SNPs are important tools for molecular genetic analysis, due to their high frequency, wide distribution [53, 54], high polymorphism, and ability to reveal fine-scale genetic variation [55]. Chen et al. (2015) showed that SNP markers are much more densely distributed than are SSR or other markers [54]. In total 1,001,405 SNPs were obtained in this study, and more than 21,371 SNPs were mapped to the chromosomes of pepper, covering the entire pepper genome more densely than in the map published by Cheng et al. (2016) [56]. The SNP coverage (more than 0.042) in our study was much higher than that in Chen et al. (2015) (~0.00208) [54], and provides adequate marker density and increases the accuracy of candidate gene identification. A total of 106,848 high-quality SNP markers were used for association analysis to identify candidate regions closely associated with FFN in pepper. To improve the accuracy of FFN candidate region identification, we overlapped results from ED association analysis and SNP-index association analysis, which accurately and quantitatively evaluated parental allele frequencies and the inheritance of parental alleles by F_2_ progenies [57]. Although the criterions for the threshold set in ED analysis and SNP-index analysis were different, candidate regions identified in ED analysis were almost in coincidence with that in SNP-index analysis, revealing the accuracy for FFN candidate region identification. As the threshold at the 99% of confidence interval was used in SNP-index analysis, the results obtained in SNP-index analysis would be more accuracy than that in ED analysis. Therefore, the final candidate regions were then narrowed down to a 3.98-Mb interval on chromosome 12 harboring 393 high-quality SNP markers. Annotations for SNP markers revealed that these SNP markers, 98% of which were located in intergenic regions, were useful for refining the mapping of a gene related to FFN to a smaller region.

Since the CM334 reference genome was released in 2014, it has been available for comparisons of high-throughput sequencing results from other crosses to determine the distribution of DNA polymorphisms across the pepper genome [21]. FFN, an important criterion of fruit earliness in pepper, is quantitatively inherited [11]. When FFN is lower and plants are shorter, fruits develop earlier [9, 10]. We mapped the FFN trait to 10 candidate regions on pepper chromosome 12 in the intervals from 198,792,663 to 198,793,573 bp, from 199,120,000 to 199,293,531 bp, from 199,293,569 to 199,952,426 bp, from 199,970,974 to 200,210,994 bp, from 200,211,315 to 200,281,965 bp, from 200,326,950 to 200,359,194 bp, from 200,629,900 to 200,638,585 bp, from 201,010,194 to 201,036,653 bp, from 201,044,155 to 201,044,589 bp, and from 201,055,482 to 203,828,248 bp. These intervals ranged from 0.00091 to 2.77 Mb in length. In previous studies, QTL for length of the primary axis and leaf number on the primary axis have also been mapped to chromosome 12 [13, 19]. Additionally, FFN is an important factor related to flowering time [16], which has also been mapped to chromosome 12 [13]. Because FFN is positively correlated with plant height, primary axis length, the number of leaves, and the number of branches [10] in pepper, loci in addition to FFN on chromosome 12 are likely important for early vegetative development, which is consistent with the results of Alimi et al. (2013) [19]. However, Alimi et al. (2013) mapped leaf number on the primary axis to a region different than that related to FFN at 23.1 cM on chromosome 12 [19]. We conclude that the 3.98-Mb interval associated with FFN on pepper chromosome 12 is a strong candidate region for FFN in pepper that could contain the gene(s) controlling this trait.

### We identified 5 candidate genes correlated to FFN in pepper

Among the 23 candidate genes for which annotations were found in the GO, COG, KEGG, and Swiss-Prot databases, some might be related to FFN, and should be useful for future gene isolation and functional testing studies. S4, S5 and S6 Figs, Fig 4 and Table 4 show details of the annotations from the GO, COG, KEGG and Swiss-Prot databases of the 23 candidate genes identified in this study that can be related to FFN. The transition of the SAM from vegetative to reproductive growth [1, 2] reveals its importance in the development of the first flower. In previous studies, *FA*, *CaJ*, *Ca-ANANTH*, *Ca-LEAFY*, *Capsicum annuum* S, and *CaBLIND* were found to promote flower development [1–5], while the *CaRNA*-binding protein and *Ca-APETALA2* were found to suppress flower formation [7, 8]. Tan et al. (2015) annotated three homologs of *Arabidopsis APETALA2* and *CLF* [14] as related to leaf number on the primary axis in pepper, which might also be related to FFN. FFN is not only relevant to flowering, but also to plant growth and development, including plant height, primary axis length, lateral branch number, and leaf number on the primary axis [9, 10]. Based on annotations in our study, we have identified five candidate genes correlated to FFN in pepper that are homologous to flower or stem development-related genes in *Arabidopsis*. *CA12g15160*, which is homologous to *Arabidopsis At1g48120* and encodes a serine/threonine-protein phosphatase 7 long form homolog, is particularly interesting because it is closely related to development of the SAM [47]. Due to the importance of the SAM in the development of the first flower, the gene *CA12g15160* is an important candidate gene for FFN in pepper. Additionally, *CA12g15390*, *CA12g15370*, and *CA12g15130* are also highly expressed in *Arabidopsis* flowers [43–46], and the GO annotation for *CA12g15360* indicates that it could be related to xylem and phloem development. The protein encoded by *CA12g15130* might take part in both the ‘phagosome’ pathway and the ‘SNARE interactions in the vesicular transport’ pathway. Although these genes that were annotated based on the reference genome are homologous to genes related to flower or stem development in *Arabidopsis*, none of previously known flowering genes have been found within the candidate regions. On the one hand, the constructed reference genome is still not perfect enough to find all the genes controlling pepper flowering due to the large pepper genome (3.48 Gb) [21]. On the other hand, BSA used in the study can be extended to identify a few major genes that controlling quantitative traits, but it is not useful to the analysis of minor genes [58]. Additionally, we still lack direct evidence that these genes control the FFN trait of pepper. Future functional analyses of these candidate genes could reveal whether they play any parts in the control of the FFN trait in pepper.

## Conclusions

In the present study, we combined the SLAF-seq technology and BSA to successfully identify genomic regions involved in control of the FFN trait in pepper (*Capsicum annuum* lines Z4 and Z5). This method proved efficient for mapping genes related to the FFN trait in the reference genome (*Capsicum annuum* line CM334). Ten candidate regions within a 3.98-Mb interval on chromosome 12 were detected and associated with expression of the FFN trait. Five out of 23 candidate genes, in particular *CA12g15160*, were chosen for further analysis based on their annotations. The functions of the genes correlated to FFN will be further examined in future studies using transformation and mutation approaches.

## Supporting information

S1 Fig SLAFs distributed on chromosomes of the pepper reference genome in the preliminary restriction enzyme digestion experiment (A), and SLAFs (B) and SNPs (C) distributed on chromosomes of samples. The *x*-axis and *y*-axis represent the length and sequence of each chromosome, respectively. Each yellow bar indicates a chromosome that is divided into 1-Mb intervals and the black line indicates SLAF or SNP.
(TIF)

S2 Fig Distribution of mapped pair-end reads in the rice genome. (TIF)

S3 Fig Circular graphic results from analysis of genome sequence variants and combined SLAF-seq and BSA association analyses in the parental lines and two bulked DNA pools. The first circle represents the 12 pepper chromosomes. The second circle represents the genes distributed along the pepper chromosomes. The third circle represents the SNP density distribution. The fourth circle represents the distribution of Euclidean distance values. The fifth circle represents the distribution of ΔSNP-index values. Data were graphed using the Circos program (http://circos.ca/).
(TIF)

S4 Fig Functional classification of candidate genes *via* Gene Ontology term analysis. (TIF)

S5 Fig The annotated ‘phagosome’ pathway associated with candidate genes in the candidate regions. Blue boxes represent all of the known enzymes that participate in the ‘phagosome’ pathway and the red box indicates the enzyme associated with the annotated match to the candidate gene.
(TIF)

S6 Fig Annotated ‘SNARE interactions in the vesicular transport’ pathway associated with candidate genes in the candidate regions. Blue boxes represent all of the known enzymes that participate in the ‘SNARE interactions in the vesicular transport’ pathway and the red box indicates the enzyme associated with the annotated match to the candidate gene.
(TIF)

S1 Table Distribution of SLAFs and SNPs on each chromosome of *Capsicum annuum* lines Z4 and Z5. (DOCX)

S2 Table The properties of total SNPs identified in samples. (XLSX)

S3 Table Annotation of SNP markers in candidate region for the parents and pools using association analysis based Euclidean distance or SNP-index. (DOCX)
