# Supplementary figures and images for: Candidate genes for first flower node identified in pepper using combined SLAF-seq and BSA
Source: PLoS One. 2018 Mar 20;13(3):e0194071. doi: 10.1371/journal.pone.0194071 (PMC5860747; doi:10.1371/journal.pone.0194071)

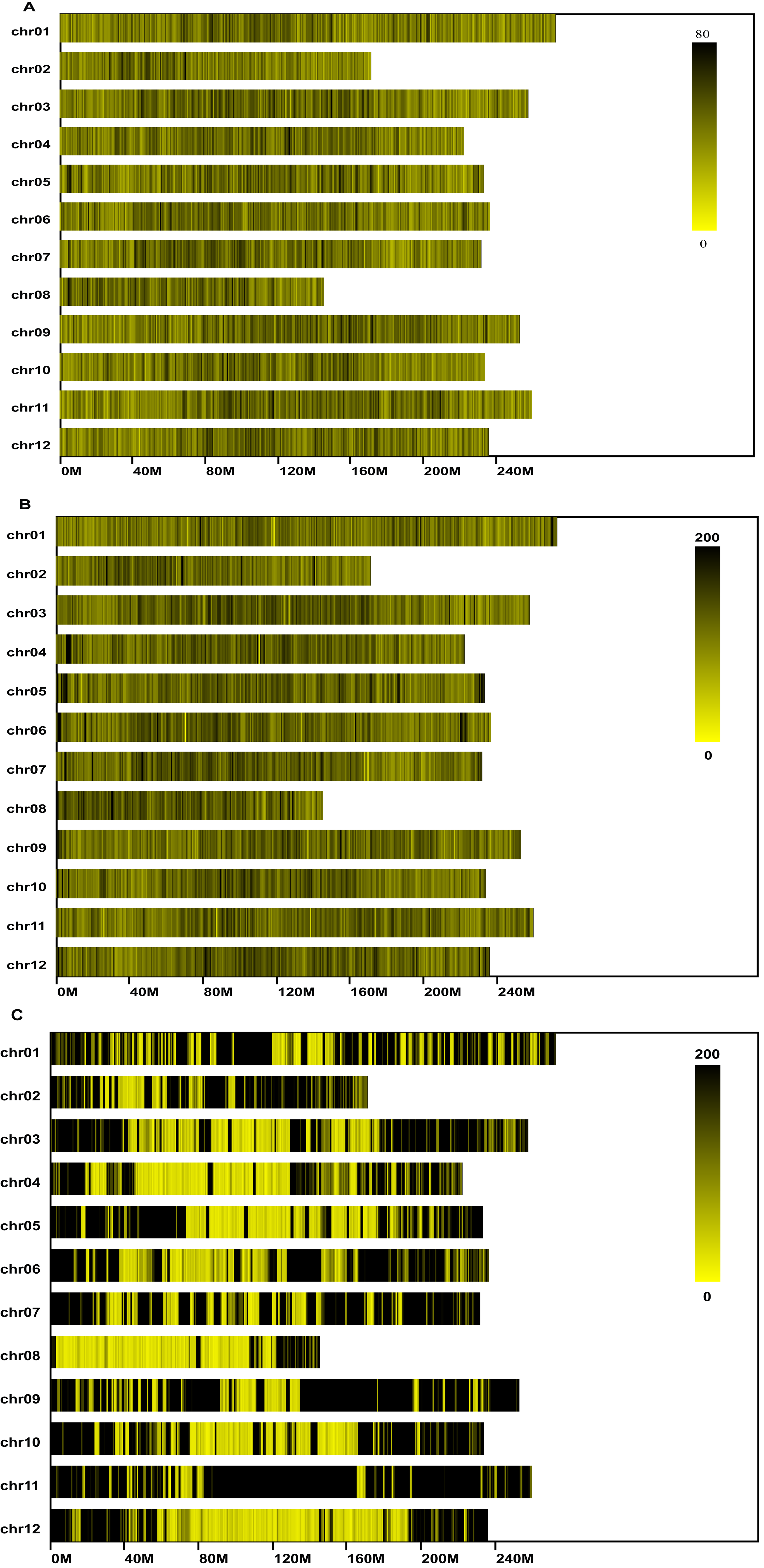

Supplement: S1 Fig — The x-axis and y-axis represent the length and sequence of each chromosome, respectively. Each yellow bar indicates a chromosome that is divided into 1-Mb intervals and the black line indicates SLAF or SNP. (TIF) [file pone.0194071.s001.tif]

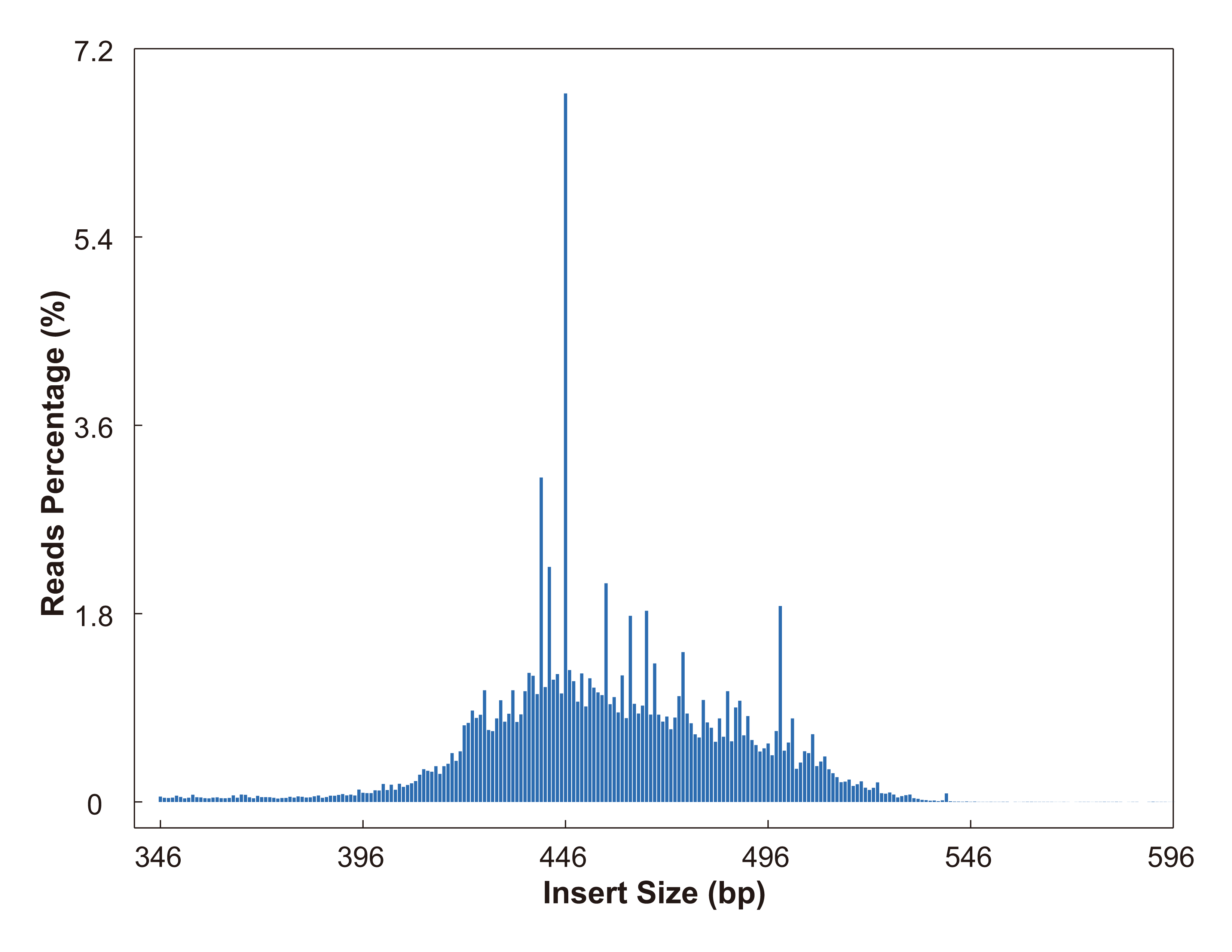

Supplement: S2 Fig — (TIF) [file pone.0194071.s002.tif]

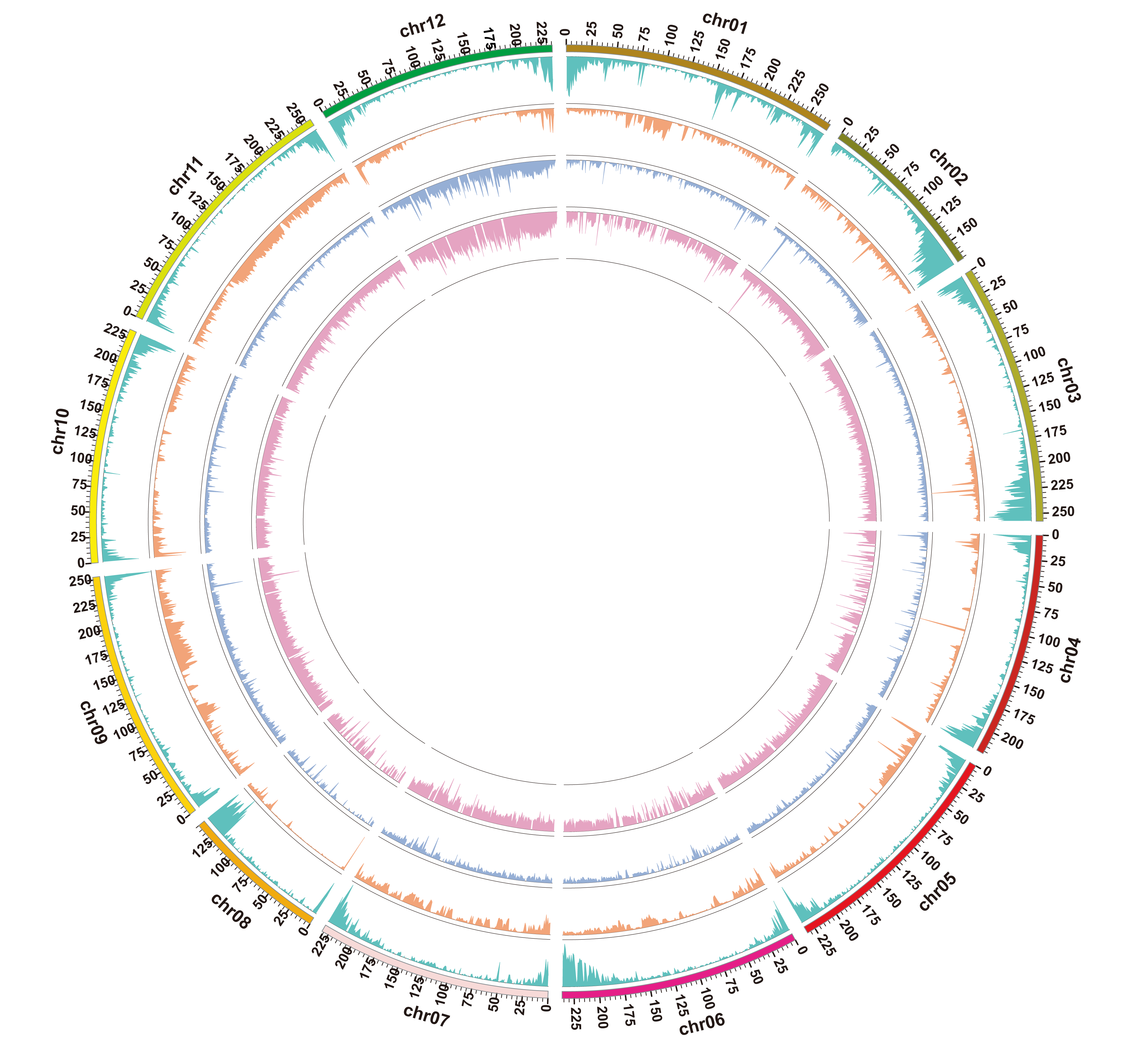

Supplement: S3 Fig — The first circle represents the 12 pepper chromosomes. The second circle represents the genes distributed along the pepper chromosomes. The third circle represents the SNP density distribution. The fourth circle represents the distribution of Euclidean distance values. The fifth circle represents the distribution of ΔSNP-index values. Data were graphed using the Circos program (http://circos.ca/). (TIF) [file pone.0194071.s003.tif]

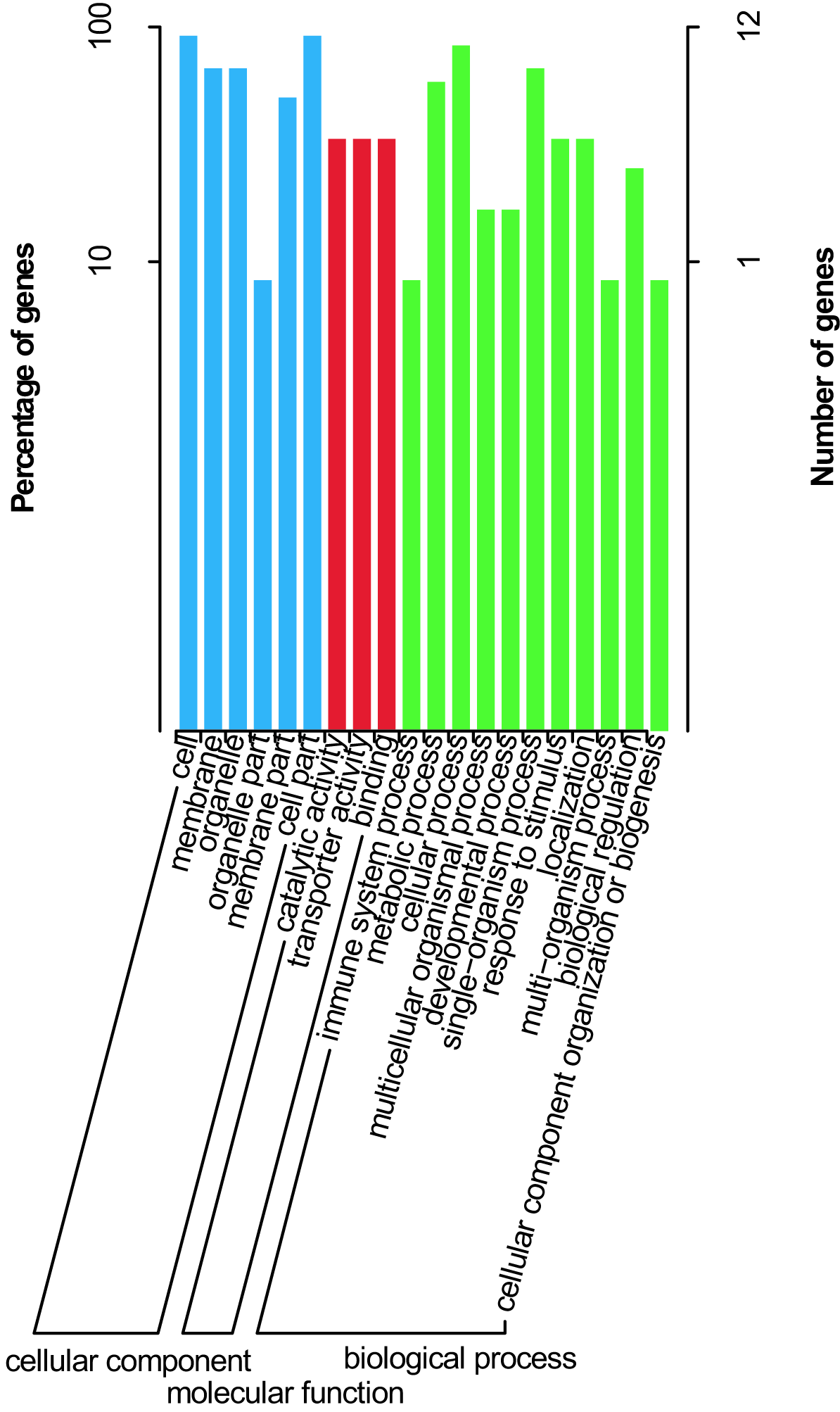

Supplement: S4 Fig — (TIF) [file pone.0194071.s004.tif]

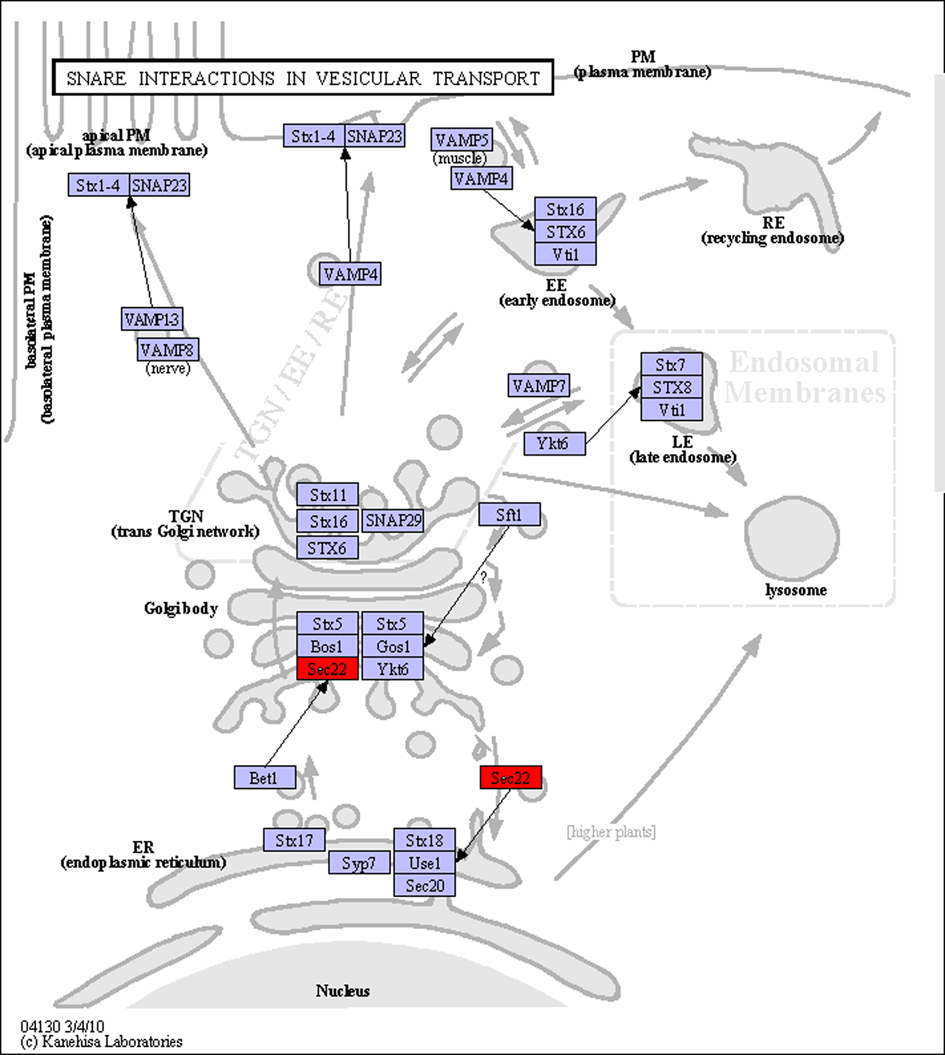

Supplement: S5 Fig — Blue boxes represent all of the known enzymes that participate in the ‘phagosome’ pathway and the red box indicates the enzyme associated with the annotated match to the candidate gene. (TIF) [file pone.0194071.s005.tif]

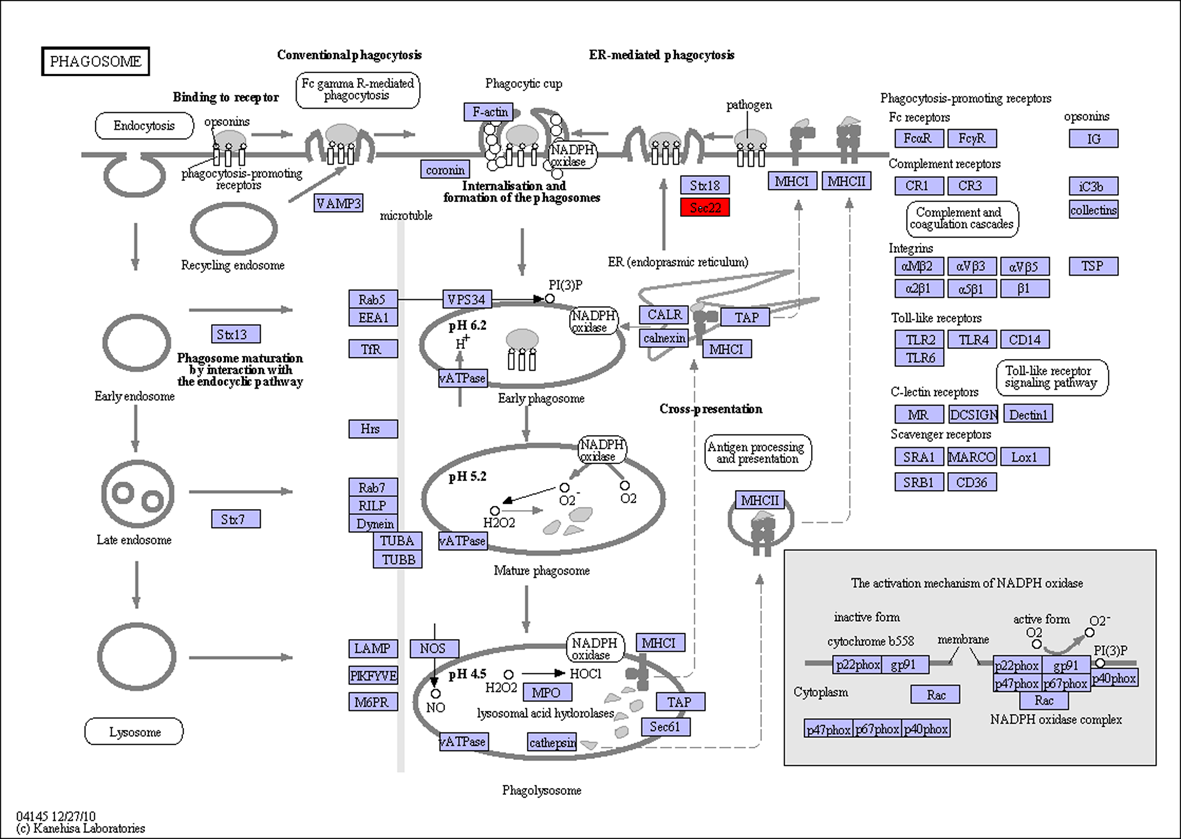

Supplement: S6 Fig — Blue boxes represent all of the known enzymes that participate in the ‘SNARE interactions in the vesicular transport’ pathway and the red box indicates the enzyme associated with the annotated match to the candidate gene. (TIF) [file pone.0194071.s006.tif]
