# Supplementary material for: Candidate genes for first flower node identified in pepper using combined SLAF-seq and BSA
Source: PLoS One. 2018 Mar 20;13(3):e0194071. doi: 10.1371/journal.pone.0194071 (PMC5860747; doi:10.1371/journal.pone.0194071)
Supplement: S1 Table — (DOCX) [file pone.0194071.s007.docx]

**S1 Table. The distribution of SLAFs and SNPs on each chromosome of *Capsicum annuum* lines Z4 and Z5.**

| **ChrID** | **SLAF number** | **SNP number** |
| --- | --- | --- |
| Chr01 | 44,917 | 105,419 |
| Chr02 | 29,871 | 60,603 |
| Chr03 | 46,254 | 76,529 |
| Chr04 | 40,544 | 51,976 |
| Chr05 | 42,249 | 77,113 |
| Chr06 | 42,812 | 74,493 |
| Chr07 | 42,335 | 98,318 |
| Chr08 | 27,872 | 21,371 |
| Chr09 | 44,815 | 149,469 |
| Chr10 | 41,783 | 74,879 |
| Chr11 | 45,748 | 150,965 |
| Chr12 | 43,059 | 60,270 |
| Total | 492,259 | 1,001,405 |

ChrID, the abbreviation of chromosome followed by a chromosome number; SLAF, specific-locus amplified fragment; SNP, single-nucleotide polymorphism.
