# Supplementary material for: Candidate genes for first flower node identified in pepper using combined SLAF-seq and BSA
Source: PLoS One. 2018 Mar 20;13(3):e0194071. doi: 10.1371/journal.pone.0194071 (PMC5860747; doi:10.1371/journal.pone.0194071)
Supplement: S3 Table — (DOCX) [file pone.0194071.s009.docx]

**S2 Table. Annotation of SNP markers in candidate region for the parents and pools using association analysis based on SNP-index.**

| **Type** | **Z4 vs Z5** | **H-pool vs L-pool** |
| --- | --- | --- |
| Intergenic | 588 | 418 |
| Intron | 0 | 0 |
| Upstream | 3 | 4 |
| Downstream | 3 | 1 |
| Synonymous coding | 0 | 0 |
| Non-synonymous coding | 0 | 0 |
| Other | 2 | 3 |
| Total | 596 | 426 |

L-pool, DNA pooled from 30 plants with the lowest first flower node value; H-pool, DNA pooled from 30 plants with highest first flower node value.
